# Virtual reality and consciousness inference in dreaming

**DOI:** 10.3389/fpsyg.2014.01133

**Published:** 2014-10-09

**Authors:** J. Allan Hobson, Charles C.-H. Hong, Karl J. Friston

**Affiliations:** ^1^Division of Sleep Medicine, Harvard Medical SchoolBoston, MA, USA; ^2^Department of Psychiatry and Behavioral Sciences, Johns Hopkins UniversityBaltimore, MD, USA; ^3^The Wellcome Trust Centre for Neuroimaging, University College LondonLondon, UK

**Keywords:** sleep, consciousness, virtual reality, prediction, free energy, rapid eye movements, pontine-geniculate-occipital waves, neuromodulation

## Abstract

This article explores the notion that the brain is genetically endowed with an innate virtual reality generator that – through experience-dependent plasticity – becomes a generative or predictive model of the world. This model, which is most clearly revealed in rapid eye movement (REM) sleep dreaming, may provide the theater for conscious experience. Functional neuroimaging evidence for brain activations that are time-locked to rapid eye movements (REMs) endorses the view that waking consciousness emerges from REM sleep – and dreaming lays the foundations for waking perception. In this view, the brain is equipped with a virtual model of the world that generates predictions of its sensations. This model is continually updated and entrained by sensory prediction errors in wakefulness to ensure veridical perception, but not in dreaming. In contrast, dreaming plays an essential role in maintaining and enhancing the capacity to model the world by minimizing model complexity and thereby maximizing both statistical and thermodynamic efficiency. This perspective suggests that consciousness corresponds to the embodied process of inference, realized through the generation of virtual realities (in both sleep and wakefulness). In short, our premise or hypothesis is that the waking brain engages with the world to predict the causes of sensations, while in sleep the brain’s generative model is actively refined so that it generates more efficient predictions during waking. We review the evidence in support of this hypothesis – evidence that grounds consciousness in biophysical computations whose neuronal and neurochemical infrastructure has been disclosed by sleep research.

## INTRODUCTION

What can sleep and dreaming tell us about consciousness (Windt and Noreika, 2011)? The answer offered in this article regards waking and sleeping consciousness as complementary and necessary for each other. This circular dependency rests upon a view of the brain as an organ of inference, generating virtual realities to explain the waking sensorium – and optimizing generative models during sleep. This view appears to be consistent with the neurobiology of sleep and the phenomenology of dreaming. This article considers the evidence that links active visual (conscious and unconscious) inference in waking and rapid eye movements (REMs) in sleep.

Our waking perception rests on a reconstruction of the world by the brain – a fantasy or hypothesis that explains sensations that are sampled from the world (Gregory, 1980). This article suggests that the capacity to model the world underlies perception in both waking and dreaming. We further propose that dreaming is a crucial prelude to waking perception. In brief, we associate conscious (and unconscious) perception with predictions of sensory inputs during waking – predictions that are generated by an internal model of the world that is embodied by the brain. In sleep, we hypothesize that this model is optimized so that it generalizes to novel situations in waking; thereby furnishing more efficient predictions and (statistical) modeling of sensory data. Crucially, this optimisation in sleep rests on the same neuronal mechanisms (i.e., synaptic activity and plasticity) that are engaged during waking perception. This provides a functional (model optimisation) and phenomenal (perceptual inference) account of dreaming. Because dreaming is highly correlated with REM sleep, the statistical and energetic functions of REM sleep may therefore inform the neurobiology of conscious and unconscious processing. REM sleep frees the brain from sensory enslavement – disclosing its constructive and integrative processes. As noted by Revonsuo (2006, p.75) dreaming “reveals consciousness in a very special, pure, and isolated form.” Our premise is that this freedom is necessary for model optimisation, during which the brain can rehearse fictive scenarios that may or may not be encountered in waking.

This article addresses the phenomenology of dreaming, the neurophysiology of REM sleep, its ontogeny, its phylogeny, and its manifestation over the life span – all of which can be interpreted under a virtual reality hypothesis. When a Helmholtzian formulation of the inferential brain is applied to these data, new statistical, and thermodynamic principles emerge, which extend the explanatory power of the virtual reality hypothesis – and may cast new light on the nature of consciousness and its disorders. We will call on promising lines of research that establish the validity of this hypothesis; particularly in relation to the inferential processes that underlie consciousness. The Ego or Self concept that emerges from this treatment owes much to the work of physiologists and philosophers who emphasize the predictive aspects of the brain in grounding (waking and dreaming) consciousness in a first-person explanation of the sensorium (Llinás and Paré, 1991; Revonsuo, 1995; Metzinger, 2009, 2013). In this view, the embodied self is a dynamic construct of the brain that is established during sleep (and *in utero*) and carried forward – fit for purpose – into waking.

Specifically, we propose that REM sleep is an occasion for reiterating and optimizing a generative model of the embodied self with reference to waking experience. This updating rests upon maximizing the statistical – and implicitly thermodynamic – efficiency of generative models of the embodied self. In other words, REM sleep is necessary to minimize their complexity. Our emphasis on the self-as-agent is based on the observation that dreams are almost always first-person. We see this fact as supporting the idea that self-model is a construct of the brain and a central part of a phenomenal experience. In other words, the self is the foundational epicenter of a world from which we sample sensations.

This article comprises two parts: in the first two sections, we overview some important phenomenological and neurobiological aspects of sleep and dreaming. In the remaining (four) sections, we lay out our theoretical framework and then try to account for the empirical facts established in the first two sections. In detail, we first review the phenomenology of dreams, with a special focus on the dreaming associated with REM sleep. We then provide a selective summary of neurobiological findings from sleep research that inform the functional anatomy of REM sleep. Our focus here is on early neurophysiological discoveries and more recent advances using brain imaging and neuropsychology. This section closes with a brief treatment of lucid dreaming that has important implications for hierarchical models in the brain. The third section introduces the Helmholtzian perspective and generative models, linking the virtual reality and Bayesian brain hypotheses. Specifically, we will look at perceptual inference in dreaming and wakefulness from the perspective of predictive coding – and the role of sleep in Bayesian model optimization. The subsequent section pursues optimization; highlighting a fundamental link between the thermodynamic and statistical efficiency afforded by Bayesian model optimization. The penultimate section reviews the empirical evidence for the ensuing role of sleep and dreaming, which is considered from a philosophical perspective in the final section.

## THE PHENOMENOLOGY OF DREAMS

In reviewing the phenomenological contrast between waking and dreaming, we will emphasize dream states associated with REM sleep, acknowledging that there are many interesting distinctions (and commonalities) between REM and non-REM sleep. Waking and dreaming have important similarities and marked differences. Chief among the similarities are the vivid and detailed perceptions and marked emotions that characterize both states. These similarities are often interpreted as *copies* of waking by dreaming but might as well be *predictions* of waking by dreaming – as we will argue. Our point here is not to stump for an either/or distinction but rather to explore the idea that waking and dreaming are both leaders and followers. Indeed, one might argue that “not only are dreams experiences but, in a way, all experiences are dreams” (Revonsuo, 2006, p.55). Dreaming tells waking what to expect and waking verifies or refutes those expectations.

Chief among the differences is the fact that dreaming is, by definition, virtual; since its subjective attributes are fabricated internally. The internal fabrication of dreams has been seen as the replay of remembered experience but this cannot be the explanation of much dream imagery, which either has no identifiable mnemonic source or may even run counter to those sources that are readily identified and easily specified in waking. It is this synthetic feature that gives dreaming its deserved pride of place in the psychology of the imagination. Dream synthesis compels us to consider the predictive and creative role that we assign to dreaming.

Other important differences between waking and dreaming are notable: waking memory is greatly enfeebled in dreaming. So is orientation, especially to time and place – as well as to third (but not first) persons. Logical inference is virtually impossible in dreaming. While emotions overlap to some extent, dreaming is associated with a narrower range and a greater depth of emotion than waking – as if dreaming were some proving ground for instinctual feelings. Movement is imagined but not actually enacted in dreaming (with the important exception of eye movements) – as if dreaming were a warm-up exercise for the game of waking.

Many of these observations come from formal analyses of dreaming phenomenology that provides direct access to anatomy of subjective processing. Formal analysis emphasizes the form of the dream as opposed to its content. Formal analysis may be preferred to content analysis (Freud, 1920; Hall and Van de Castle, 1966) in the study of how virtual realities are generated by the brain. To clarify the distinction between formal and content analysis, we will present and analyze a specimen dream recorded by one of us (AH) using both its form and content:

**Execution dream** (9/23/2011): sometime after 3 AM I had the following vivid dream:

I was in a very large and elegant apartment (not mine or any other place that I recognize).

*There was to be a series of two or maybe three stereotyped killings that I had arranged or at least approved. Each killing was the shooting, by long rifle, of a victim unknown to me but who deserved to die. In each case, the murder was accomplished by a couple (again people unknown to me). I could clearly see the victim and the murderous couple; feel satisfaction as one member of the couple, usually the man, took careful aim at his victim who did not flinch from fear or apprehension. I don*’*t recall ever hearing a shot, or seeing any victim fall but the series of executions was carried out efficiently at my behest. I was mildly surprised but very satisfied to see it all go so well.*

When this had gone on for what I would estimate to be about 5 or maybe 10 min, I began to feel frightened. What if the police came? Would I be arrested?

Suddenly I myself was holding a gun. The weapon was a poorly made rifle with the stock and barrel fastened together with crude screws and nuts. I reasoned that I could dismount this weapon and hide its parts. But where? The police would surely look under the mattresses that I considered as possible hiding places. I asked myself, what if the police also realize that I had engineered an earlier crime (of which I was sure I had dreamt before).

### FORMAL ANALYSIS (FIRST PERSON)

In terms of its form, this is a typical REM sleep dream with vivid percepts (McCarley and Hoffman, 1981), delusional belief (Hobson et al., 2000), cognitive defects (Hobson and Stickgold, 1994; Stickgold et al., 2001), and both indifference and intense feeling (Merritt et al., 1994). My perceptions were detailed and hallucinatory with strong and clear visual imagery. I never once suspected that I was dreaming, always supposing, erroneously, that I was awake. I was disoriented – having no idea where I was, how I got there, or where I might go next.

In terms of the suggestion that dreaming reinforces the sense of self-as-agent, it is important to stress that I never assumed that I was anyone but me in the dream, yet I had no idea who my hired assassins might be or who the victims were – or even why they were victims. This brutal aggressiveness did not bother me at all in the dream. Other emotions, such as anxiety and apprehension, were strong once I realized what had transpired. My thought processes were grossly impaired, especially with respect to the (almost) complete loss of critical self-reflective awareness and insight.

In the second part of the dream I *did* begin to worry about the consequences of the executions, confirming the assertions of Kahan and LaBerge (2010) that self-reflective awareness is never completely absent from dreams. I was holding a makeshift gun and wondering how to dismantle and hide it lest my part in the murders be discovered by the police. It seems likely that this shift to a more rational, detached observational and reflective stance in the second part of the dream would be accompanied by frontal lobe activation and a reciprocal weakening of REM physiology – as seen in lucid dreaming (Voss et al., 2009).

### CONTENT ANALYSIS

A dream content analyst or therapist might well be interested in my murderous impulses and how they relate to my life. Content-wise, this was an unusual dream for me. In my dreams as in my waking life, I am usually engaged and ecstatic, with positive social interaction and affect. I am almost never the dispassionate, detached voyeur of this particular dream scenario. But, I must admit this was my dream – so I need to take responsibility for its content

Many of the formal properties of dreams can be reliably recognized and measured, allowing subjective experience to be correlated with physiological or behavioral responses: for example, in sensory perception (Hong et al., 1997, 2009; Horikawa et al., 2013), visual scanning (Roffwarg et al., 1962; Herman et al., 1984; Hong et al., 1995), motor control (Dresler et al., 2012), language (Hong et al., 1996), multisensory binding (Llinas and Ribary, 1993; Hong et al., 2009), and the organization of intrinsic brain networks (Koike et al., 2011). Furthermore, considerable experience has been accrued using sleep lab reports and home-based data (Hobson, 1988, 1999, 2002; Hobson and Stickgold, 1994; Kahn and Hobson, 1994, 2005; Resnick et al., 1994; Rittenhouse et al., 1994; Stickgold et al., 1994a,b, 2001; Sutton et al., 1994a,b).

In Freudian dream analysis, content is the manifest and latent content of a dream as it is remembered and its hidden meaning respectively, (Freud, 1920). More recent empirical approaches to content analysis have been pioneered by Hall, on the basis of thousands of dream reports. Empirical categories, such as “characters” and “social interactions,” were subsequently refined and elaborated by (Hall and Van de Castle, 1966). Most elements in a dream report some fit into one or more categories (e.g., hugging someone is a friendly interaction and a physical activity). Content categories can then be used to create indicators that pertain to the dreamer or cohorts of dreamers. See Schredl (2010), for a contemporary review of content analysis in sleep research.

Virtual reality dream theory asserts that we explore a potentially infinite repertoire of predictive dream scripts. These scripts or scenarios are rehearsed in dreaming to provide an efficient (minimally complex) portfolio of explanations for the waking sensorium. However, the key thing we need to explain is why the phenomenology of dreams is so (apparently) delusional, hallucinatory and formally disordered. We will return to this issue after reviewing the neurobiology of sleep and theories of dreaming.

## A SELECTIVE HISTORY OF SLEEP AND DREAM SCIENCE

The discovery of brain activation in sleep by Aserinsky and Kleitman (1953) marked a turning point in the history of consciousness science. The (REM sleep) brain activation they described was qualitatively similar to that of waking – as was the subjective experience of dreaming. These similarities suggested an intimate relationship between brain function in sleep and waking that we will consider in later sections. First, we will look more closely at the nature of REM sleep.

### THE NEUROBIOLOGY OF REM SLEEP

The association of brain activation with REMs suggests an endogenous (brain stem) source of neuronal activation – and its role in the internal generation of visual imagery in dreaming. The description of REM sleep muscle atonia by Jouvet and Michel (1959) helped to presage the idea that dreaming was constituted by virtual perception and movement. Real (waking) perception and movement were actively blocked, while the brain was forced to generate a virtual simulacrum of waking consciousness. As the physical basis of the most intense dreaming, REM sleep is generally taken to be the canonical physiological substrate of dream consciousness.

The Aserinsky-Kleitman discovery led to a series of (largely futile) attempts to test psychoanalytic theory, which then dominated the intellectual scene. Because Michel Jouvet was a neurosurgeon, not a psychiatrist or psychoanalyst (and French, not American), he quickly exploited the biological significance of his discovery and proceeded to localize REM sleep control to the pontine brain stem (Jouvet, 1962) and to attempt to characterize REM genesis in neurochemical terms (Jouvet, 1969, 1972). Thus Jouvet was able to add modulation (M) to the already strong evidence for activation (A) and input–output gating (I) – three functions later used to construct a quantitative AIM model of conscious brain states (Hobson et al., 2000).

Other biologically grounded work showed that REM occurred in most mammals and in young birds (Klein et al., 1964; Roffwarg et al., 1966; Allison and Cicchetti, 1976). REM was significantly overrepresented in immature mammals and birds – and was even more evident with prematurity (Dreyfus-Brisac, 1964). At 30 weeks of gestational age, the percent of REM time has been estimated at a hundred percent (Roffwarg et al., 1966; Dawes et al., 1972; Birnholz, 1981). A neurodevelopmental function for REM could therefore be safely assumed.

These facts suggested that to support abundant and vigorous REM sleep, an animal needed to be large-brained and young. These conclusions are relevant to virtual reality dream theory, since they indicate both a neurodevelopmental function and a synthetic strategy for consciousness. Of course, REM sleep is not only present in young developing animals. Rather, its persistence over the life span hints at continued instantiation of intrinsic neuronal programs and the continuous modification by experience. The evidence that sleep favors learning is extensive and REM sleep in particular enhances motor skill acquisition (Walker et al., 2002; Diekelmann and Born, 2010). An intriguing discovery was that mammals and birds that evinced REM were not only dreamers but thermoregulators as well. Crucially, this thermoregulatory (homeothermic) capacity is lost during REM sleep. These discoveries raise interesting questions; for example, what is the functional evolutionary imperative of REM sleep that compensates for the suspension of homeothermy (Hobson and Friston, 2012) – and what is the relationship between thermodynamic and computational costs entailed by the brain activity during REM sleep. We will address these questions later.

### THE NATURE OF REM SLEEP AND DREAMING

Many have emphasized the virtual and predictive aspect of dreaming and dream consciousness. For example, Llinás and Paré (1991) considered the idea of dreaming and waking consciousness as predictive models of the world. For example, in their treatment of dreaming and wakefulness they conclude: “These considerations lead us to challenge the traditional Jamesian view of brain function according to which consciousness is generated as an exclusive by-product of sensory input. Instead, we argue that consciousness is fundamentally a closed-loop property, in which the ability of cells to be intrinsically active plays a central role” (Llinás and Paré, 1991).

In a similar vein, Revonsuo presented a Virtual Reality metaphor of consciousness arguing “that the subjective form of dreams reveals the subjective, macro-level form of consciousness in general and that both dreams and the everyday phenomenal world may be thought of as constructed virtual realities” (Revonsuo, 1995). It was later proposed that dreaming is a preparatory, predictive simulation in the Threat Simulation Theory (Valli et al., 2005). Thomas Metzinger and Jennifer Windt have analyzed and refined the idea of dreaming as a model or simulation of the world that transcends waking and dreaming consciousness: “what we call waking life is a form of online dreaming” (Metzinger, 2003; p. 140). From this mix of comparative neurobiology and philosophy grew our concept of protoconsciousness and the virtual reality hypothesis (Hobson, 2009) – under which sensorimotor integration was supposed to be inborn and develop *in utero*. This concept accommodates the fact that dream consciousness *precedes* waking consciousness by months in utero and hours in adult life – it emphasizes self-as-agent, self-acting in a virtual (exteroceptive) space. Later, we will consider implicit virtual reality models as the precursor of generative models; namely, the models that emerge when the brain engages with the job of explaining exteroceptive input (after birth or sleep). We now turn to a promising area of research that has revealed much about the functional anatomy of sleep.

### NEUROIMAGING

Brain imaging has revolutionized sleep research and is starting to elucidate the neurobiology of consciousness. Over the past two decades, positron emission tomography (PET) of human subjects has revealed REM sleep activation, above the level of waking, in the following regions:

- The *pontine tegmentum* (Maquet et al., 1996; Braun et al., 1997) confirming the results of Jouvet (1973) in the cat.
- The *amygdala* (Maquet et al., 1996; Braun et al., 1997; Nofzinger et al., 1997) and the *parahippocampal cortex* (Braun et al., 1997; Nofzinger et al., 1997) in keeping with the emotionality of dreaming (Merritt et al., 1994).
- The *parietal operculum* (Maquet et al., 1996; Braun et al., 1997; Nofzinger et al., 1997) a cortical hub for associative integration (Hobson and Stickgold, 1994).

Complementing these positive findings is the singular and unexpected observation of persistent deactivation of the frontal cortex (specifically the dorsolateral prefrontal cortex, Braun et al., 1997; Nofzinger et al., 1997). Note that this is consistent with the cognitive defects revealed by formal analyses of dreams.

The findings of brain imaging are surprisingly consistent and robust. They confirm – and extend to man –neurophysiological findings in experimental animals. As such, they carry with them the theoretical implications already noted from basic research. It seems quite likely that the cellular and molecular mechanisms of brain activation are the same across mammalian species, including humans. These cardinal neuroimaging results are also consistent with brain lesion studies, which we now touch upon.

### THE NEUROPSYCHOLOGY OF DREAMING

The neuropsychologist Mark Solms interviewed and obtained computerized axial tomography (CAT) data from several hundred human subjects admitted to a London hospital. His findings complement the PET data: a complete loss of dreaming was reported by victims of stroke damage to either the occipito-temporo-parietal junction or deep frontal white matter. Furthermore, a remarkably astute examination of lobotomy subject records uncovered reports of complete loss of dreaming following surgery.

Thus it seems reasonable to hypothesize (Solms, 1997) that frontal projections are crucial to dreaming (although instrumental awakenings may be required to ensure that dreaming itself – and not just the recollection of dreaming – is impaired by brain lesions). Solms also affirms Epstein (1977) and the PET amygdala activation (Maquet et al., 1996; Braun et al., 1997; Nofzinger et al., 1997) by establishing a possible contribution of the temporal lobe to the hyperemotional, fugue-like and automatic quality of dreams (see dream phenomenology). The temporal lobe is notoriously seizure prone (Epstein, 1977) and its REM sleep brain activation by ponto-geniculo-occipital (PGO) wave excitation (Calvo et al., 1992) is relevant to the hypothesis of a seizure-like process in normal sleep.

### LUCID DREAMING

Dreams, such as the specimen dream above, are normally delusional as to the state of consciousness in which they unfold. The dreamer supposes him or herself to be awake when he or she is, in fact, fast asleep. This delusion is easily resolved by awakening the dreamer, whose brain then becomes a more accurate (sensory-bound) instrument. The delusion can also be recognized and dispelled by introducing waking awareness into the dream. This creates lucidity, which can be defined as the conscious (doxastic) awareness that one is dreaming instead of believing, falsely, that one is awake.

Lucid dreaming is of central importance to the virtual reality hypothesis, because it clearly demonstrates the validity of three foundational assumptions:

(1) There are two states of consciousness: one is waking and the other is dreaming.
(2) Waking and dreaming consciousness are normally separate and distinct, but,
(3) They can coexist as a hybrid state, in which both are present.

The hybrid state of lucid dreaming – however, rare and evanescent – can be scientifically investigated as shown by Steven LaBerge, whose pioneering sleep lab studies proved that lucid dreaming – a first-person experience – always arises out of REM sleep and can be identified by a third person observer (LaBerge, 1990). Similarly, Ursula Voss has demonstrated that waking, non-lucid and lucid dreaming have quantitatively distinct EEG power signatures and that frontal lobe activation was lowest in non-lucid dreaming, highest in waking, and intermediate in lucid dreaming (Voss et al., 2012). Finally, Martin Dresler used fMRI to show that a distinct brain circuit was activated in lucid dreaming – a circuit that might mediate this curious state (Dresler et al., 2012).

The upshot of this work is that the brain mediates at least three states of consciousness, each in precise and distinctive ways. The crucial variable here is activation: both regional and global brain activation can be spontaneously and voluntarily changed to enhance one state of consciousness or another. The incidence of spontaneous lucidity peaks at about age nine (Voss et al., 2012), while volitional lucid dreaming can be cultivated most easily in young adults, under the age of thirty (Hobson, 2010).

Why age should be so crucial in determining dream lucidity is a question for future investigation. Most studies of dreaming have been conducted using young adult subjects – a limitation easily overcome using journalistic and home-based recording methods (Stickgold et al., 2001). Another interesting question concerns the apparently divided “self” of lucid dreamers: one watches, while the other dreams. Furthermore, the watching self can influence dream continuation and command changes in dream content. We speak of “being of two minds” and this is literally true in the case of lucid dreaming. We also speak of “talking to ourselves” – often in an encouraging way – as if we were indulging in autosuggestion.

Emergence of the observing self is associated with frontal lobe activation (Voss et al., 2009), a fact that accords well with executive functions attributed to this region. We know that dreaming is a brainstem-posterior forebrain affair (Jouvet, 1973; Maquet et al., 1996) and that frontal lobe activation is suppressed in REM (Braun et al., 1997; Nofzinger et al., 1997). The functionally “split brain” of sleep may thus become a rich source of data for studies of volition and metaconsciousness.

Having reviewed the functional anatomy of REM sleep and dreaming, we now turn to more formal models of conscious perception. Our hope is to explain the neurobiology and phenomenology of the dreaming in theoretical terms that call on generative models of the world.

## VIRTUAL REALITY AND THE BAYESIAN BRAIN

Our thesis is that dreaming and REM sleep may hold the key for understanding the nature of conscious inference and its neurophysiological underpinnings. This understanding rests upon viewing the phenomenology of dreams and their neurophysiology in terms of perceptual synthesis – using a generative model of the sensorium. This section considers the special role that dreaming has in optimizing virtual reality models in the brain. In brief, we propose that dreaming and REMs are natural consequences of model optimization – in which the model is optimized to minimize redundancy or complexity. This will become crucial from the point of view of metabolism and energy efficiency – and its association with sleep – considered in the next section.

In what follows, we will refer to the brain as performing inference. Inference is usually thought of as reasoning on the basis of evidence; for example, inferring the probability of rain when deciding to take a raincoat. Almost universally, inference can be cast in terms of Bayesian inference, in which prior beliefs (April showers are common) are combined with sensory evidence (dark clouds over the horizon) to produce a posterior belief (it is likely to rain). This posterior belief is the expectation that maximizes Bayesian model evidence. We will take inference to be any process that increases the Bayesian model evidence associated with posterior expectations. Crucially, this inference may or may not be consciously articulated – but can always be defined mathematically and functionally.

### THE BAYESIAN BRAIN

The notion that perception occupies the realm of a virtual reality dates back to Plato and his allegorical cave – in which are sensory impressions were likened to shadows cast by firelight on a cave wall. Perception makes sense of these impressions and, necessarily, entails some form of inference or modeling. This was most clearly articulated by Helmholtz (1866/1962) in terms of unconscious inference and the generation of explanations for sensory impressions:

*“Objects are always imagined as being present in the field of vision as would have to be there in order to produce the same impression on the nervous mechanism*”

– von Helmholtz

Over the last century, this insight has been treated in several guises – from Gregory’s notion of perception as hypothesis testing (Gregory, 1980) to current formulations of the Bayesian brain (Knill and Pouget, 2004). They all appeal to an underlying generative model from which predictions or hypotheses are drawn. One might ask what why we equate (phenomenal) virtual realities with (inferential) generative models.

The concept of virtual reality is often used to refer to the subjective experiences of a self immersed in a world that appears real (Revonsuo, 1995, 2006). The generative models that underlie the Bayesian brain hypothesis may, at first glance, fail to emphasize the subjective aspect of virtual reality model. However, virtual reality and generative models are formally equivalent – in the sense that generative models generate predictions of sensory contact with a world that is perceived through sensations. However, there is an important distinction between virtual reality and generative models: the purpose of a generative model is to account for (sensory) data. This means that when exposed to sensory information, the generative model – embodied by the brain – is trying to recapitulate reality through sensory exchanges with the world. It is therefore constrained by reality. Only when asleep, is the brain freed from sensory constraints to generate fictive predictions or a virtual reality proper. In short, we may be born with a virtual reality model but sensory exchanges with the real world quickly mould it into a generative model – with nightly reprises from predicting sensations during sleep. Note that the generative model is quintessentially subjective or first-person because the sensory predictions are unique to the self-as-agent in a sampled world.

The most neurobiological plausible instance of this predictive sampling is known as predictive coding; particularly of a hierarchical sort (Srinivasan, Laughlin and Dubs, 1982; Rao and Ballard, 1999; Bastos et al., 2012; Clark, 2013; Hohwy, 2013). Predictive coding involves the updating of expectations about hidden states of the world generating sensory data. This updating is driven by sensory information that cannot be explained by current expectations or beliefs entertained by the brain. This information is known as prediction error and is simply the difference between sensory input and top-down or descending predictions of that input. When generalized to hierarchical models, the same reciprocal exchange of descending top-down predictions and ascending or bottom-up prediction errors emerges. This provides a nice metaphor for the recurrent message passing between levels of the cortical hierarchy – and accounts for many anatomical and physiological aspects of extrinsic (long-range) connections in the brain (Lee and Mumford, 2003; Friston, 2008; Bastos et al., 2012). The idea here is that prediction errors inform expectations at higher levels of the hierarchy so that they can send better predictions to lower levels – and thereby suppress prediction error. If one can suppress prediction error at all levels of the cortical hierarchy, then the implicit expectations – encoded neuronally – provide a plausible explanation for sensory input at multiple levels of description or abstraction.

Clearly, this reciprocal message passing or hierarchical predictive coding rests upon a model that can generate top-down predictions. It is this hierarchical model – entailed by cortical hierarchies – which we associate with the virtual reality model of dream theory. The principle that drives recurrent neuronal exchanges is to minimize prediction error and thereby make (Bayes) optimal predictions that underlie perceptual *inference* or synthesis. In this setting, minimizing prediction error corresponds to maximizing Bayesian model evidence. Exactly the same prediction error minimization underlies changes in synaptic efficacy – through associative or experience-dependent plasticity – enabling the brain to acquire models of increasing hierarchical depth. This is referred to as perceptual *learning*.

There is now a large body of circumstantial evidence for predictive coding in the brain, both in terms of hierarchical cortical architectures and the canonical microcircuits required to construct predictions (Bastos et al., 2012; Adams et al., 2013a). In brief, current thinking suggests that prediction errors are encoded by firing rates of superficial pyramidal cells in the upper layers of the cortex. The predictions, in turn, are conveyed by top-down or descending backward connections from deep pyramidal cells encoding expectations or beliefs about (hidden) states in the world causing sensory impressions (Mumford, 1992; Bastos et al., 2012). But what has this view of neuronal processing to do with sleep and dreaming?

To understand this, we have to step back from predictive coding – and the minimization of prediction errors – and think about the underlying quantity that is being optimized during perception. From a statistical perspective, this quantity is the (Bayesian model) evidence for the generative model. In other words, the model is optimized or adjusted until the probability of sensations – over an extended period of time – is the most probable under all models that could be entertained. Practically, Bayesian model evidence is very difficult to compute, so a proxy is generally used both in statistics and – we suggest – the brain. This proxy is called variational free energy (Hinton and van Camp, 1993; Beal, 2003), leading to the variational free energy formulation of perceptual inference and learning (Dayan et al., 1995; Friston et al., 2006).

From the point of view of sleep, the key thing to appreciate is that maximizing model evidence (or minimizing variational free energy) does not just rest upon minimizing prediction errors but also requires that they are minimized as parsimoniously as possible. Technically speaking, the (logarithm of the) Bayesian model evidence can be decomposed into *accuracy* and *complexity*; where log evidence increases with accuracy (small prediction errors) but decreases with complexity – the degrees of freedom required to make predictions (Penny et al., 2004). This means that there is an imperative to minimize the complexity of generative models to maximize their evidence.

### COMPLEXITY AND SLEEP

Complexity is a measure of how complicated a generative model is. In other words, complexity reflects the degrees of freedom – or numbers of parameters – that are required to provide an accurate prediction of sensory data. Minimizing complexity under the constraint of maintaining accurate predictions is nothing more than the principle of Occam’s razor; formulated in terms of Bayesian model selection (Penny et al., 2004). Technically speaking, complexity is measured as the difference between prior beliefs (expressed as a probability distribution) and posterior beliefs – after the prior beliefs have been updated on observing sensory outcomes. We have previously discussed the physiological basis for this complexity reduction in terms of eliminating redundant parameters or synapses during sleep (Hobson and Friston, 2012). This is exactly consistent with the synaptic homoeostasis hypothesis and the role of sleep in pruning unnecessary and exuberant synaptic connections (Gilestro et al., 2009). This pruning minimizes the redundancy (complexity) of the model and endows it with a parsimony and hierarchical simplicity that is essential for efficient and generalizable perceptual inference during waking (i.e., precludes overfitting). In summary, the very notion of a model induces the concept of model complexity and an associated cost function that has to comply with Occam’s principle. Physiologically, we have suggested that sleep is necessary to minimize complexity in order to ensure that our models are optimal during waking. But what is special about sleep from point of view of model optimization?

According to the AIM hypothesis, REM sleep is the quintessential state of perceptual processing in which the brain is sequestered from sensory perturbations. This modulatory input gating is crucial for minimizing complexity – because we can ignore the accuracy part of model evidence (because there are no sensations for which an accurate explanation is required). This means that the brain can focus on minimizing complexity, using exactly the same hierarchical prediction error minimization that it uses during wakefulness. Indeed, statistical schemes that operate along these lines can be found in powerful machine learning algorithms such as the wake-sleep algorithm (Hinton et al., 1995).

Notice here, it is the synaptic connections *learning* associations and contingencies that are optimized, not the synaptic activity *inferring* current states of the world. In other words, brain activity is freed from explaining sensory input and is used to generate fictive predictions that produce prediction errors at each level of the hierarchy. These prediction errors then drive activity-dependent plasticity to minimize complexity (by minimizing prediction error). We have here the first glimpse of a functional explanation for the formal phenomenology of dreams. The distinction between inference and learning is central to our arguments – and the distinction between waking and dreaming perception. Inference corresponds to optimizing neuronal or synaptic activity at a fast timescale over hundreds of milliseconds, while learning corresponds to a slow optimization of synaptic connections over minutes to hours. In waking, inference is enslaved by sensory input and learning is driven vicariously by (experience dependent) plasticity. In contrast, during sleep there is no sensory entrainment – and learning (synaptic plasticity) can only reduce model complexity; in other words, make the generative model internally consistent. This means the perceptual content of dreams – encoded by synaptic activity – is fictive in the sense that it corresponds to real-world scenarios that never actually occur. Put simply, the content of dreams is not a prediction of what will happen but an exploration of what could (or could not) happen that is necessary to minimize model complexity – rendering it a more efficient model of the experienced world on waking.

### COMPLEXITY MINIMISATION AND DREAM PHENOMENOLOGY

In the absence of sensory constraints, the vivid percepts (McCarley and Hoffman, 1981), delusional beliefs (Hobson et al., 2000) and cognitive defects (Hobson and Stickgold, 1994; Stickgold et al., 2001) cease to be delusional or defective – because these attributes are only defined in relation to sensory evidence. However, in sleep, there is no sensory evidence and the only imperative is to adjudicate and select among unconstrained scenarios that can be entertained by the sleeping brain. The implicit (protoconscious) self-supervision may also explain frontal deactivation and the temporary suspension of top-down constraints on lower-level representations – representations or beliefs that compete for “synaptic resources.” As noted above, this functional hypofrontality may preclude the veridical self-awareness and doxastic attributions that characterize dreaming.

In this view, dream content corresponds to expectations or beliefs encoded by endogenous neuronal activity that both causes, and is caused by, changes in synaptic connectivity. Both neuronal activity and connectivity minimize prediction errors, even in the absence of sensory input – because prediction errors are prevalent throughout the depth of cortical and subcortical hierarchies. The only difference between dreaming and waking is that the precision (neuromodulatory gain) of prediction errors at the lowest (sensory) levels and deepest (executive) is reduced – leaving intermediate levels to sculpt themselves into a parsimonious and self-consistent architecture, that will be more efficiently entrained by the waking sensorium.

Interestingly, Jesse Prinz (2000) has argued that there is something special about processes that operate upon “intermediate-level representations.” A natural speculation is that inferences that are geared to action-selection predominate at intermediate levels – apt for the selection and control of embodied action. We will pursue this in setting of oculomotor control below. However, the notion of intermediate cortical levels generating a virtual reality – that is freed from both (bottom-up) sensory constraints and (top-down) contextual constraints from prefrontal narratives – may also have something to say about dream content.

If the purpose of dreaming is to simplify our model of the world, why are dreams so diverse and, if anything, more complex than the world itself? Hofstadter (1979) touches on this issue: by exploring themes common to the works of the logician Gödel, artist Escher and composer Bach, Hofstadter discusses how self-reference allow systems to generate meaning. His treatment rests on recursion and self-reference, in a way that is very reminiscent of hierarchical inference and reciprocal message passing in the brain. Hofstadter asserts that dreams are unpredictably diverse and infinitely creative. How does this fit with complexity minimisation? The key thing to note is that complexity is an attribute of a model, not the content of (fictive) inferences afforded by a model. As noted above, the function of complexity minimisation is to ensure that the model can generalize. In other words, minimizing the complexity of synaptic connections enables efficient inferences about a greater diversity of sensory scenarios – scenarios that may be rehearsed during dreaming. It is the very diversity of dream content that complexity minimisation aspires to accommodate. In this sense, dreaming may prepare the brain for the unpredictable diversity of scenarios it encounters during waking. Complexity minimisation therefore furnishes a plausible perspective on the diversity of dream content but what about its creative aspects?

Clearly, freed from the top-down (prefrontal) constraints of narratives and perspective-taking, dream content can take on a fantastical aspect that can entertain violations of temporal and perspectival contiguity. But how does this relate to creativity? A potential answer to this question may lie in daydreaming and mind-wandering (McMillan et al., 2013). Several lines of research have linked mind-wandering to creativity, especially in problems that have been encountered recently (Baird et al., 2012). Using an incubation task and a validated creativity task – the Unusual Use Task – Baird et al. (2012) showed that creative problem solving can be enhanced by performing an undemanding distraction task during an incubation period (c.f., eureka moments that come out of the blue). In short, the ability to realize creative (generalizable) associations rests on a temporary suspension of cognitive and attentional set – of the sort associated with the prefrontal cortex (Christoff et al., 2009). This fits comfortably with the suspension of high level (prefrontal) constraints that we suppose helps optimize associative connection strengths during sleep.

In summary, our explanation for the function of sleep suggests that the brain is essentially doing the same thing in sleep and waking; with one key difference – there is no sensory input during sleep. However, the recurrent hierarchical message passing is still in process; with continually changing expectations and hierarchical predictions that constitute dream content. Physiologically, it appears that cholinergic discharges enable the promulgation of ascending prediction errors at intermediate levels of the cortical hierarchy, while aminergic neuromodulation suppresses or gates ascending sensory input at the sensory level (Hobson and Friston, 2012). This means that, from the brain’s point of view, the world is still unfolding with deep hierarchical structure and perceptual content – the only thing that has changed is that this content is no longer enslaved by sensory information.

Having said this, the brain is still compelled to minimize (extrasensory) prediction errors and implicitly complexity. Heuristically, one can imagine how this proceeds by considering prediction errors as reporting inconsistent or over-parameterized models. As the models are revised during dreaming, different hierarchical representations become internally consistent producing – on average – fewer hierarchical prediction errors. The endpoint of this process is a hierarchical model that can generalize to the diversity of sensory scenarios it encounters during waking. This offers an explanation for the function of sleep. But how does it explain the physiology of sleep?

### ACTIVE INFERENCE AND RAPID EYE MOVEMENTS

According to the AIM model, the transitions from wakefulness to sleep rest upon a selective gating of sensory inputs that is entirely consistent with the neurochemistry of the wake-sleep cycle (Hobson, 2009). The selective pressure for this diurnal gating can now be motivated in terms of model optimization or complexity minimization. Furthermore, the advantage afforded by parsimonious but hierarchically deep models may explain the association between REM sleep and higher levels of consciousness in comparative sleep research (see above and Hobson, 2009).

One interesting aspect of this formulation is the special status of eye movements during sleep. All perceptual inference is at some level active – in the sense that we actively sample our sensorium to create our own sensations (Wurtz et al., 2011; Brown et al., 2013). This embodied perspective is referred to as *active inference* (Friston et al., 2011). This is important because higher levels of the model are necessarily producing multilateral top-down predictions of both visual (and other exteroceptive) input *and the proprioceptive consequences of actively sampling that input*. Indeed, under active inference, the descending proprioceptive predictions become motor commands that are fulfilled by peripheral reflexes in the spinal-cord and pontine nuclei (Adams et al., 2013a). In this formulation, classical reflexes simply minimize (proprioceptive) prediction error.

This is important because – unlike the striatal muscle of the motor plant – it is possible to move the eyes without changing posture. In other words, proprioceptive predictions descending within the central nervous system can engage the oculomotor system with impunity. In turn, this means model optimization during sleep (and *in utero*) can include predictions about visual and oculomotor sensations that engage the full depth of the hierarchy – spanning the visual (geniculo-occipital) system and the (pontine) oculomotor system. Physiologically, this means that we would expect to see classical oculomotor reflexes fulfilling proprioceptive predictions – and producing eye movements in sleep that are not dissimilar to saccadic eye movements. At the same time, descending visual predictions will fall upon deaf ears (sic) as they encounter the (gated) sensory level at the lateral geniculate or early visual cortex. This provides a simple explanation for REMs in REM sleep and – electrophysiologically – for their association with PGO waves.

In summary, the generative or virtual model entertained by the brain requires maintenance – in the sense it has to account for a vast amount of sensory input during waking and can only do this if it generalizes to every context encountered. This generalization rests upon minimizing model complexity (to avoid overfitting sensory data), which is equivalent to minimizing variational free energy in the absence of sensory input. The neurophysiological validity of variational free energy minimization – in terms of predictive coding and associative plasticity – has already been established in terms of neuronally plausible mechanisms (Friston et al., 2006; Bastos et al., 2012). Furthermore, we have discussed at length the role of sleep in enabling this optimization (Hobson and Friston, 2012). This perspective provides a simple and mechanistically grounded account of the neuromodulatory gating of cortical and subcortical systems, the selective expression of eye movements in sleep and their association with PGO waves. In the penultimate section, we will expand upon the role of virtual reality models in conscious inference. In the next section, we consider the relationship between complexity minimization, energy regulation and sleep.

## REM SLEEP, MODEL COMPLEXITY AND THERMODYNAMIC EFFICIENCY

We have hinted at an intimate association between homoeothermic regulation and REM sleep in terms of comparative physiology. There are some further fascinating links between thermoregulation and REM sleep that we now pursue in light of complexity (variational free energy) minimization during sleep.

A remarkable fact about REM sleep is that homeothermy is suspended and temperature sensitive neurons in the hypothalamus become temperature insensitive in REM (Parmeggiani, 2003). The predictive coding formulation above provides a simple explanation for the implicit loss of temperature control during sleep: if aminergic modulation suppresses the sensitivity of principal cells reporting sensory (interoceptive) prediction errors, then it will preclude the signaling of thermoreceptors along unmyelinated C-fibers and delta-fibers. In short, the brain will be impervious to fluctuations in temperature and will not respond to suppress thermal prediction errors, resulting in a suspension of homeothermy. So what evolutionary imperatives endorse this risky physiology? The answer that emerges from the above arguments is that sleep is an optimization process that is disclosed by the nightly removal of sensory perturbations; in other words, the brain can take itself off-line with impunity, so that synaptic plasticity and homoeostasis (Gilestro et al., 2009) can reduce the complexity it has accrued during wakefulness. In evolutionary terms, the adaptive cost of nightly suspensions of homeothermy is offset by the reduction of complexity costs afforded by sleep – provided environmental temperature does not fluctuate too much during sleeping (or *in utero*).

As noted in Hobson and Friston (2012), the imperative to reduce complexity during sleep may be greater for the (complicated) brains of mammals (and birds). The failure to restore complexity to minimal levels would, in principle, mean that experience-dependent learning during the day would not be tempered; leading to a colloquial and context-bound model of the world that becomes increasingly complex and redundant. In statistics, the equivalent pathology is known as “over-fitting” and leads to suboptimal models that fail to generalize beyond the data on which they were trained. In short, taking the brain off-line to prune exuberant associations established during wakefulness may be a necessary price we pay for having a sophisticated virtual reality model that can distil complex and subtle associations from the sensorium. But is this the complete story?

In fact, there is a more fundamental link between complexity minimization and thermoregulation that provides a thermodynamic perspective on minimizing complexity costs. The arguments here are subtle but simple (a detailed discussion can be found in Sengupta et al., 2013). The premise is that minimizing complexity implicitly maximizes the thermodynamic efficiency of information processing in the brain. This premise can be verified by noting that when the brain minimizes complexity it also minimizes its thermodynamic free energy (and the work needed to attain that state). In short, a minimally complex brain state is also in an energetic minimum:

Any system – including the brain – will minimize its *thermodynamic* free energy when isolated from external forces or perturbations. However, when isolated from sensory perturbations, a system that is trying to minimize model evidence (or *variational* free energy) will minimize complexity – which means the state of minimum complexity is also the state of minimum thermodynamic free energy. More generally, simpler models are less costly both in terms of statistical or model complexity and the thermodynamic work entailed by applying them to (sensory) data. Quite literally, our might brains run a tiny bit hotter after a sleepless night – as they try to inefficiently over-fit sensory data.

The notion – that a failure to minimize information-theoretic free energy in sleep entails a failure to minimize thermodynamic free energy – seems borne out by the fate of Rechtschaffen et al.’s (1989) rats (Jim Hopkins – personal communication). As Rosalind Cartwright reports (Cartwright, 2010, pp37–8) these sleep-deprived rats died as a consequence of metabolic burn-out. This apparently resulted from a failure to lower (or down-regulate) their core body temperature (NREM and REM sleep had different effects but REM deprivation alone was sufficient). The mechanisms remain unclear but – in the present context – speak to a failure of (interoceptive) predictive coding, resulting (fatal) failures to minimize both information theoretic and thermodynamic free energy.

Note that we are not saying the sleeping brain finds some equilibrium steady-state of minimum energy consumption. Rather, we are saying that the drive toward simpler models implicit in synaptic regression – and other physiological mechanisms – during sleep, renders the brain’s non-equilibrium steady-state functioning less metabolically expensive when averaged over both sleep and wakefulness. In the next section, we will consider neuroimaging evidence that large portions of the brain reduce their metabolic rates during REM sleep – but other regions show activation during REM sleep. Interestingly, the particular systems showing REM-locked activation are those involved in intermediate hierarchical representations and the enabling of message passing among those levels.

In summary, we have seen that the imperative to minimize model complexity is equivalent to optimizing the thermodynamic efficiency of information processing in the brain. Given that the previous section established sleep as necessary for complexity minimization, it follows that sleep is also necessary to optimize the metabolic efficiency of operating the virtual reality model in wakefulness. We now consider empirical evidence for the theoretical considerations above, with a special focus on the neuronal systems generating and searching fictive visual scenes.

## EMPIRICAL EVIDENCE FOR PREDICTIVE CODING DURING SLEEP

In this section, we review recent functional magnetic resonance imaging and electrophysiological evidence that is consistent with the computational architecture implied above. Our focus is on the correlates of REMs and the modulation of fast synchronous (gamma) activity by (cholinergic) gating mechanisms in REM sleep.

### REM-LOCKED ACTIVATION IN THE BRAIN

We recently reported an fMRI study of the neural correlates of REMs in sleep (Hong et al., 2009). REMs were identified from video recordings that detects about four times as many REMs than conventional electrooculographic (EOG) approaches that are usually used in fMRI studies (Wehrle et al., 2005; Miyauchi et al., 2009). This is because removing MRI scanner artifacts from the EOG also removes small amplitude eye movements. In contrast, video monitoring reveals small eye movements, which is important because both small and large eye movements can be detected with fMRI (Kimmig et al., 2001).

Our key findings can be summarized as follows: REM-locked activation is distributed but regionally specific. Peak activation is clearly localized in primary visual and non-visual sensory cortex and regions implicated in perceptual binding or synthesis: namely, the thalamic reticular nucleus, claustrum, and basal cholinergic forebrain. The reticular nucleus of the thalamus and claustrum have been identified as structures that are crucial for binding information distributed within and across different sensory modalities (Crick, 1984; Crick and Koch, 2005). The role of the claustrum in binding and salience detection has also been considered by Smythies et al. (2012) and Remedios et al. (2010), respectively. Furthermore, electrical stimulation of the claustrum has been shown to produce reversible arrest of volitional behavior and unresponsiveness (Koubeissi et al., 2014). REM-locked activation was also found in multisensory cortical areas indentified in waking studies (Calvert and Thesen, 2004). These include superior temporal gyrus – a key region for audiovisual integration (Hein and Knight, 2008) – and the right retrosplenial cortex.

REM-locked activation overlaps with brain regions expressing or modulating gamma oscillations (Gross and Gotman, 1999; Jouny et al., 2000); i.e., the thalamocortical sensory system (Llinas and Ribary, 1993), mesopontine tegmentum (Steriade et al., 1991) and basal forebrain (Szymusiak, 1995; Mesulam, 2004; Perry and Perry, 2004). These findings are consistent with a role for REMs in hierarchical multisensory integration that may be evident before birth (Hobson, 2009) and through the life span (Bremner et al., 2012).

### NEUROMODULATORY GATING AND ELECTROPHYSIOLOGICAL OSCILLATIONS

The basal forebrain cholinergic system can induce gamma band synchronization, which is a potential mechanism for the binding of distributed neuronal processes (Engel and Singer, 2001) – and regional enhancement of cortical sensory processing (Szymusiak, 1995; Mesulam, 2004; Perry and Perry, 2004). From the perspectives of the AIM model and predictive coding, cholinergic modulation may play an important role in modulating and gating prediction errors signals – a role that has been associated with the deployment of attention in waking (Feldman and Friston, 2010) and the neurochemical modulation of hierarchical cortical processing during sleep (Hobson and Friston, 2012).

Crucially, the rapid integration or binding of distributed neuronal groups is thought to be required for conscious experience. It has been proposed that “rapid integration is achieved through the process of reentry, the ongoing, recursive, highly parallel signaling within and among brain areas” (Tononi and Edelman, 1998). This fits comfortably with the reciprocal message passing mandated by predictive coding. Furthermore, the REM-locked activation of brain regions commonly associated with binding and high-frequency (gamma) synchronization is consistent with the cholinergic boosting of superficial pyramidal cells at intermediate levels in the cortex during REM sleep. Typically, superficial cortical layers show greater coherence and activity in the gamma range; for example, the superficial layers of cortex show neuronal synchronization and spike-field coherence predominantly in the gamma frequencies, while deep layers prefer lower (alpha or beta) frequencies (Roopun et al., 2006; Maier et al., 2010; Buffalo et al., 2011). This is potentially important because – as noted above – superficial pyramidal cells are thought to encode prediction error (Bastos et al., 2012).

Rapid eye movement-locked activation in the primary sensory (olfactory or somatosensory) cortex does not necessarily correspond to an olfactory or somatosensory dream experience (Hong et al., 2009). In dreaming, people rarely smell (Zadra et al., 1998) or feel touch (McCarley and Hobson, 1979). Instead, REM-locked multisensory recruitment may reflect top-down priming of sensory cortex (Tallon-Baudry and Bertrand, 1999; Calvert and Thesen, 2004; Hong et al., 2009).

REMs are accompanied by PGO waves (Nelson et al., 1983). REM-locked multisensory recruitment suggests that top-down predictions – eliciting REM and PGO waves – involve visual and non-visual components of the cortical hierarchy (Hobson and Friston, 2012). Furthermore, brain activation time-locked to REM (and PGO waves) is widespread; as predicted by the activation-synthesis hypothesis (Hobson and McCarley, 1977). However, while this activation is distributed, it is also highly system-specific, engaging exactly those systems that are deployed for hierarchical perceptual synthesis during visual palpation in waking.

In short, these results are compatible with virtual reality dream theory in that they demonstrate the activation of the brain in REM sleep recapitulates that of waking. Given the phenomenology of dreams, this is no great surprise – supporting the idea that dreaming and waking mirror each other. Instead of arguing that dreaming precedes rather than follows waking, one might argue that waking and dreaming are two sides of the same coin – interacting in a complementary and reciprocal fashion. Our two states of consciousness are mutually enhancing rather than divisively competitive.

### DO REMs SCAN DREAM IMAGERY OR GENERATE IT?

A correlation between the density of REMs in sleep and reports of visual experience in dreaming may indicate that REMs “scan” the dream scene (Roffwarg et al., 1962; Herman et al., 1984; Hong et al., 1995; Hong et al., 1997). Alternatively, it may indicate that REMs play a role in generating dream images (Hong et al., 2009). This issue is important because, in active inference, eye movements are both cause and consequence of perception. This is meant in the sense that a percept entails both visual and proprioceptive predictions – and the latter induce movement through oculomotor reflexes in sleep (REM) and wakefulness (saccades).

Are REMs driven by dream imagery or do they generate it (or both)? Studies in human subjects suggest that REMs are visually targeted eye movements – commanded by the forebrain – in response to visual dream images (Roffwarg et al., 1962; Herman et al., 1984; Hong et al., 1995, 2009). In contrast, single cell recording studies in animals have established that PGO waves that are coupled with REMs (Nelson et al., 1983). PGO waves have been interpreted as corollary discharges associated with brain stem oculomotor commands. We originally proposed that this discharge might be associated with the generation of visual images in dreams (Hobson et al., 2000). Indeed, from the perspective of predictive coding, this corollary discharge corresponds to descending visual predictions (of a virtual scene) that are an integral part of perceptual inference – inference that determines dream content.

In short, two lines of research that initially appeared to contradict each other now converge on the view that REMs are involved in both the scanning and generation of dream imagery (Hong et al., 2009). In other words, eye movements are both cause and consequence of perceptual content – a notion that is becoming increasingly dominant in the visual neurosciences. This “active vision” perspective (Wurtz et al., 2011) is consistent with active inference during waking, where perceptual content is used to generate top-down predictions of oculomotor sensations and their consequences. This provides a nice model for saccadic eye movements that are essential for waking perception (Yarbus, 1967) and sequential updating of sensory samples (Friston et al., 2012). Saccadic searches of the visual world in wakefulness are necessary for testing models of the world and serially updating the ensuing hypotheses with data sampled through scanning. This has been simulated in terms of active inference (Friston et al., 2012) – and has been pursued in the context of abnormalities seen in schizophrenia (Adams et al., 2012). Recordings of saccadic eye movements show that they are attracted to parts of the visual scene that have salient or precise information; e.g., the eyes and lips (Yarbus, 1967). These saccadic searches are performed automatically and quickly: about four to eight eye movements per second, which is interestingly about the same frequency of PGO waves and bursts of REM in sleep. It therefore appears that both waking saccadic searches and REMs are an integral part of active vision (Wurtz et al., 2011) – in much the same way that we infer the nature of an object in the dark by palpitating it.

However, there is no visual world to be scanned by a fetus. REM sleep is nonetheless preponderant in the third trimester of pregnancy. How can we interpret this fact? The answer is simple: although there are no precise visual (exteroceptive) consequences of movement, there are proprioceptive consequences that have to be learnt – and can be learned *in utero*. If this observation is right, REMs would be essential to enable the brain to model the (proprioceptive) consequences of eye movements that will be necessary during active visual searches. Crucially, REM sleep deprivation in immature rats suggests that endogenously generated visual activation during REM sleep plays a necessary role in development of the visual system (Shaffery et al., 2002).

Waking imagery studies (Brandt and Stark, 1997; Laeng and Teodorescu, 2002) suggest that information pertaining to saccadic sequences is maintained together with the visual representation – and is used as spatial index for the proper arrangement of image components during image generation (in dreaming) and perceptual synthesis (in waking). Again, we conclude that REMs are involved both in saccadic searches of the dream image and in its generation. In the setting of active inference, REMs are the peripheral expression of top-down proprioceptive predictions based upon active visual palpation of a virtual scene generated in the cortical hierarchy. It is therefore sensible – from the virtual reality perspective – that dream perception and its proprioceptive manifestation share a common visual theme.

Further evidence supports the view that REMs are involved in the generation of a virtual reality. First, REM-locked activation is greater in the posterior left hemisphere (Hong et al., 2009) that appears to be crucial for generation of dream imagery (Farah, 1984). Second, REM-locked activation in language areas speaks to the involvement of association (semantic) cortex (Hong et al., 2009) and its reciprocal interaction with lower (sensory) cortex (e.g., Tallon-Baudry and Bertrand, 1999). Finally, REM-locked activation is seen in primary somatosensory cortex, premotor cortex, vestibular cortex, insula, anterior cingulate cortex, putamen, and superior temporal gyrus (Hong et al., 2009). These regions have all been implicated in representing the embodied self (Blanke, 2012) – suggesting that REMs may be involved in the generation of (bodily) self images in dreaming.

### SOME CLUES FROM ELECTROPHYSIOLOGY

There are two key neurobiological candidates for modulating synaptic gain that are implicated in both REM sleep and attentional gating: synchronous gain (Chawla et al., 1999) mediated by fast oscillatory or synchronized activity (Womelsdorf and Fries, 2006) and classical neuromodulatory (e.g., cholinergic) neurotransmission (Schroeder et al., 2001; Hirayama et al., 2004). Furthermore, both of these gating mechanisms influence each other: as noted above, gamma oscillations are profoundly affected by acetylcholine, which is released into sensory cortex from nuclei in the basal forebrain. Acetylcholine acts through both fast ion channel (nicotinic) receptors and slow metabotropic (muscarinic) receptors (Hasselmo and Giocomo, 2006). Acetylcholine appears to increase synaptic gain directly by, for example, reducing spike-frequency adaptation. It may also facilitate the induction of gamma oscillations by reducing adaptation in pyramidal cells or decreasing activity of inhibitory interneurons (Börgers et al., 2005).

Human intracranial EEG shows that cortical gamma power is enhanced during phasic REM sleep (but not tonic REM sleep associated with generalized atonia). Similarly, rat hippocampus shows increased gamma synchrony during phasic REM sleep, while gamma power and firing rates decrease during tonic REM sleep (Montgomery et al., 2008). Therefore, gamma power may be preferentially expressed during phasic REM sleep, enabling message passing among intermediate levels of the cortical hierarchy (and explaining the relatively low gamma–REM association found by Le Van Quyen et al., 2010).

Interestingly, there is reduced EEG coherence in the gamma band between cortical areas during REM sleep (Castro et al., 2013). This is not inconsistent with the functional disconnection or suspension of top-down constraints implied by the exploratory nature of fictive predictive coding during complexity minimization. Indeed, “The virtual absence of gamma frequency coherence during REM sleep may underlie the unique cognitive processing that occurs during dreams, which is principally a REM sleep-related phenomenon” (Castro et al., 2013).

Another important electrophysiological hallmark of predictive coding is the mismatch negativity (MMN), elicited as an event related potential difference by oddball or deviant stimuli. These responses have often been associated with prediction errors. There is some evidence that an analog of the MMN can be elicited in REM sleep “despite the gross suppression of both executive top-down processing and external input transmission (Atienza et al., 2000; Ibáñez et al., 2009)” (Yordanova et al., 2012). Interestingly, no MMN has been recorded during non-REM sleep and slow wave sleep (Yordanova et al., 2012), suggesting that there may be something special about the activated brain state in REM sleep that enables descending predictions (necessary to produce the mismatch response).

In summary, much of the empirical evidence pertaining to the neurophysiology of REM sleep – as obtained through invasive and non-invasive studies – implicates the functional anatomy that underlies perceptual synthesis and binding. Furthermore, many of the neurophysiological correlates of REMs in sleep are consistent with neuronal implementations of predictive coding based on virtual reality or generative models. In particular, the involvement of brain systems that underlie active visual inference account for both the prevalence of visual content in dreaming perception and the prominence of REMs – and their associated PGO waves. In the next section, we consider the implications of the virtual reality model for understanding conscious inference – and the special role that dreaming may play in disclosing the anatomy of consciousness.

## VIRTUAL REALITY DREAM THEORY AND CONSCIOUSNESS

There are clear echoes in active inference of Kant’s search for the *a priori* conditions for the possibility of experience (Kant, 1999/1781). The prenatal evidence of highly organized brain activity of the fetal brain *in utero* (Birnholz, 1981) is a cogent of modern findings in this regard. The post-natal prevalence of REM sleep is also relevant but it could be argued that neonatal infants have an abundance of novel experiential data to assimilate into their generative models during sleep. In both cases, it seems likely that important statistical and thermodynamic processes are at work at this point in neurodevelopment.

We have already shown that several issues in consciousness research (Chalmers, 1996; Wegner, 2003) can be addressed when the brain is treated as an organ of inference (Hobson and Friston, 2014). In what follows, we briefly review how appealing to the process of inference, implicit in the deployment of virtual reality models, may provide plausible answers to metaphysical questions.

### CONSCIOUS AND UNCONSCIOUS INFERENCE

In the preceding sections, we have seen how the virtual reality model underlies inference in the brain – and how statistical imperatives persist during sleep. The neurobiological generation and refining of perceptual fantasies during dreaming was considered in light of predictive coding and the neuromodulatory gating effects of sleep. The previous section reviewed the empirical neurophysiological evidence in support of this formulation. Here, we return to our assertion that sleep and dream research can cast light upon some aspects of consciousness.

In Hobson and Friston (2014), we put forward the simple argument that consciousness can – at some level – be equated with inference. The idea here is that the dualism implied by the *res cogitans* and *res extensa* (realm of the mind and realm of extensive physical reality: Manuel, 2001, p. 97) can be resolved through the biophysical act of inference; namely, neuronal message passing in the sleeping and waking brain. This dual aspect monism follows from the fact that the biophysical state of the brain encodes probabilistic beliefs that minimize prediction error (or variational free energy). In other words, the dynamics of synaptic activity and efficacy are driven by quantities (variational free energy) that are functions of beliefs (probability distributions) encoded by those biophysical quantities. For example, population activity at the neuronal level may encode the expectation or mean of a Gaussian probability distribution over some hidden state of the world. The very fact that dynamical forces on the physical brain are produced by (functions of) probability distributions links the physical (*res extensa*) to the mindful (beliefs or *res cogitans*) in a fundamental way. Another perspective on this bridge over the Cartesian divide is that it provides a (wide sense) realization relationship (Wilson, 2001; Gillett, 2002). In other words, the process of inference affords a unique mapping between physical (neuronal) states and the properties (probabilistic beliefs) they realize (c.f., Bechtel and Mundale, 1999).

In short, we cast perception in terms of inference, where inference is associated with a distinctive functional property or role (i.e., hierarchical message passing). This functional role is realized by neuronal populations that encode probability distributions. This makes this theory a functionalist theory, which links mental to physical states as roles to realizers. As far as metaphysics goes, this is then a theory that is consistent with (i) a thoroughgoing reductive physicalism, if we identify mental states with the physical states that realize the roles, or (ii) a non-reductive physicalism, if all and only physical states play the functional roles (in the actual world), and/or (iii) with a property dualism, if realizers and roles are considered distinct properties. Property dualism is the modern type of dual aspect theory.

Many of the interesting insights offered by equating consciousness with the process of inference rest on the hierarchical nature of generative or virtual reality models. In hierarchical models, inference can be decomposed into multiple levels, with progressively higher or deeper levels of representational abstraction or explanation. This leads to the distinction between inferences at low levels of sensory hierarchies – that can be associated with unconscious inference in the sense of Helmholtz (1866/1962) – and at higher levels that could be associated with conscious percepts and concepts.

To illustrate the importance of hierarchical inference consider a concrete example, starting at the lowest level of the hierarchy; namely, a reflex. A peripheral reflex counters deviations from a proprioceptive equilibrium point set by descending corticopontine or corticospinal projections. One can associate this equilibrium point with the set point of a thermostat. From a statistical perspective, the neuronal activity encoding the equilibrium point corresponds to the expected (or mean) proprioceptive input and the gain of peripheral motor neurons encodes the inverse variance (or precision) (Adams et al., 2013a). Does this neuronal encoding of a probability distribution constitute consciousness? In the sense that it encodes a probabilistic belief, one might argue that even a simple knee-jerk reflex embodies some form of unconscious (motor) belief. However, things get more interesting if the descending proprioceptive predictions (motor commands) arise from a higher hierarchical level with autonomous dynamics (e.g., a central pattern generator). At the higher level, one can interpret neuronal activity (and gain) as encoding expectations (and precision) of movement trajectories, framed in terms of proprioceptive input.

These are beliefs about motion that entail both past and future; immediately freeing beliefs from the instant in time that they are fulfilled. Consider now a further hierarchical level that predicts (and selects) the particular trajectory that is enacted. This level may generate top-down predictions of proprioceptive trajectories and their visual consequences. In other words, we have moved beyond simple motor representations to a hierarchical level where expectations (neuronal activity and their associated beliefs) are quintessentially sensorimotor in nature. At this level, the multimodal nature of descending predictions (aka corollary discharge) renders the expectations amodal. Would these constitute conscious experience?

One could argue that these high-level, dynamically structured beliefs are much closer to phenomenal consciousness. Furthermore, if we now equip our hierarchical model with models that distinguish between the consequences of self-made acts and the acts of others, we start to get closer to conceptual expectations of the sort that may underlie subjective consciousness. Crucially, at all hierarchical levels, the biophysical drives underlying neuronal activity are physically lawful in that they minimize variational free energy – in exactly the same way that gravitational forces conform to Hamilton’s principle of least action.

### HIERARCHICAL INFERENCE AND DREAMING

The notion of hierarchical representation comes to the fore in the context of dreaming. First, we have the hierarchical distinction between sensory and higher levels. This is important because modulatory gating during REM sleep has opposing effects on the two levels – effectively suppressing the sensory level and augmenting message passing among higher levels through selective activation of ascending cholinergic systems. This means that Bayesian belief updating is implicit in the sleeping brain in a way that is formally equivalent to predictive coding of (non-gated) sensory input during wakefulness. However, the nature of conscious inference is fundamentally different in the sense that beliefs – encoded by neuronal activity and plasticity – are unconstrained by sensory information, permitting fantastical constructions.

Second, in the setting of hierarchical models, each hierarchical level or system makes inferences about others. This is exactly the conclusion reached from analysis of the phenomenology of lucid dreaming. Recall from above that the notion of watching oneself implies a partition of consciousness into the “watcher” and the “watched.” This is consistent with a hierarchical decomposition of inference during sleep. It could even be argued that the same hierarchical decomposition or meta-representational interpretation applies during wakefulness – and emerges as meta-cognition (awareness of being aware).

**FIGURE 1:**
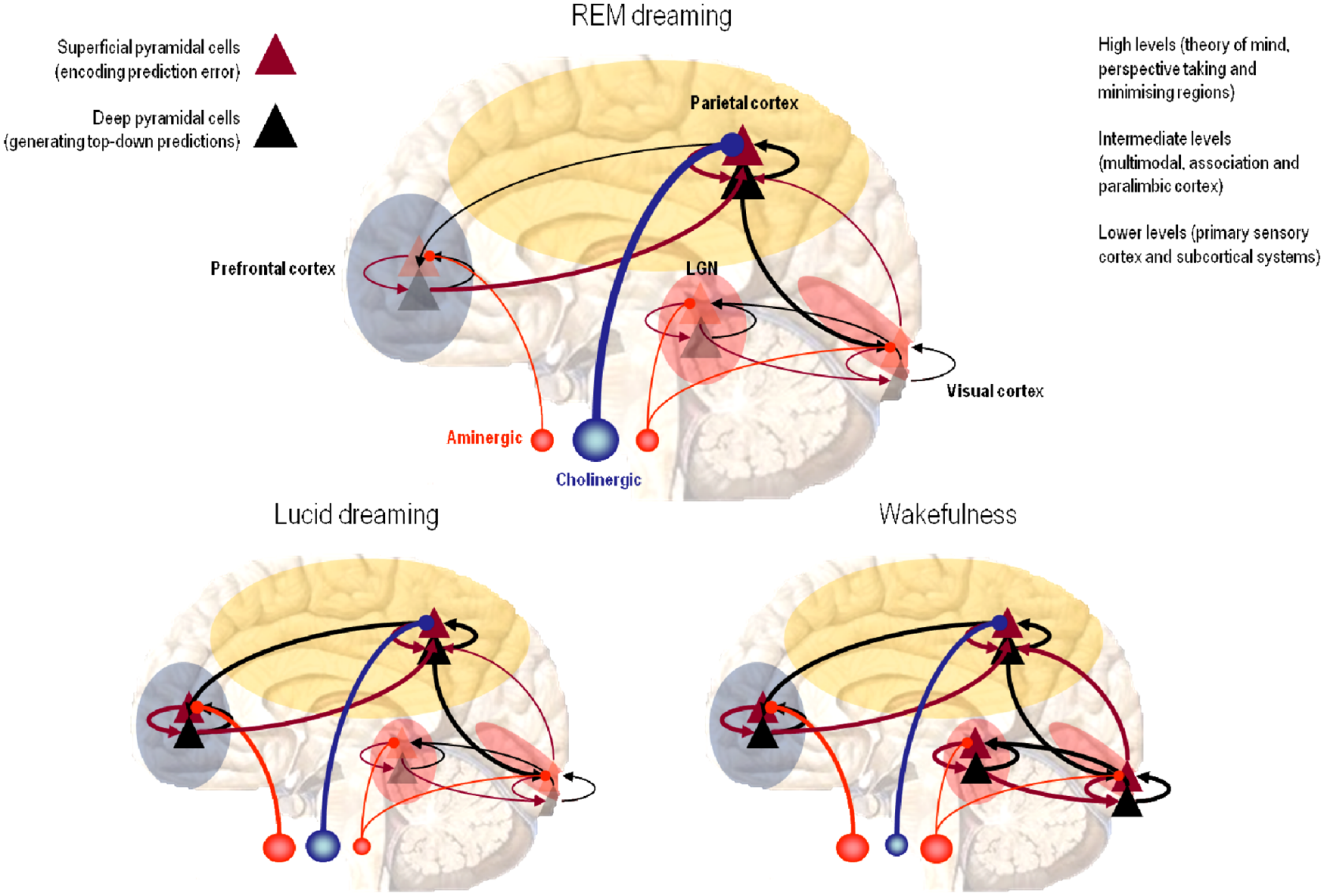
**The schematic illustrates the neuromodulatory gating of hierarchical message passing in the brain during rapid eye movement (REM) sleep **(top)**, wakefulness **(right)**, and the hybrid state of lucid dreaming **(left)**.** The anatomy of this schematic should not be taken too seriously: it is just meant to differentiate between different levels of the cortical hierarchy in terms of low (sensory) levels, intermediate (extrasensory and multimodal) levels and – for the purposes of this paper – high (meta-representational) levels. Here, we have associated higher levels with theory of mind areas that are engaged in mentalizing and perspective taking tasks. Within each level we have depicted representative cortical microcircuits in terms of superficial (red triangles) and deep (black triangles) pyramidal cells. In predictive coding formulations of neuronal message passing, superficial pyramidal cells encode prediction error that is passed up the hierarchy to update the activity of deep pyramidal cells encoding expectations (red connections). These reciprocate top-down predictions that are compared with the expectations by prediction error units in the level below (black connections). In REM sleep, the idea is that cholinergic modulation (blue projections) of superficial pyramidal cells at intermediate levels of the cortical hierarchy preferentially enables these levels, while suppressing ascending prediction errors from primary sensory cortex. In lucid dreaming, aminergic (e.g., dopaminergic: pink projections) neuromodulation sensitizes prediction errors in the prefrontal cortex, enabling top-down predictions from the highest or deepest levels of the hierarchy – endowing processing in intermediate levels with a narrative or context. In waking, aminergic (e.g., noradrenergic) neuromodulation boosts sensory prediction errors that are now able to entrain hierarchical inference in higher cortical levels for perceptual synthesis.

Recently, it has been shown that transcranial alternating current stimulation, at low gamma frequencies, induces lucidity and self-reflective awareness in REM dreaming (Voss et al., 2014). The authors assume that “lower gamma band activity is mediated by activation of fast spiking interneurons that are known to generate gamma oscillations.” The ensuing oscillations have been proposed to increase the synchronous gain of neuronal message passing. This suggests that lucid dreaming may be associated with an increase in the precision of prior beliefs (in prefrontal cortex) that underlie personal narratives (Kiebel et al., 2008) – priors that are quiescent in normal REM sleep. We again see the importance of neuromodulation in contextualizing the relative contribution (of higher, intermediate and sensory) hierarchical processing in the induction of conscious states. See **Figure 1**.

In summary, the inferential perspective on conscious processes creates a hierarchically composed theater for conscious experience that accommodates the distinction between waking and dreaming consciousness (through a dissociation of sensory and non-sensory hierarchical levels) and dissociation within higher levels that would be necessary for lucid dreaming. Within this framework, many of the formal characteristics of perception and cognition during dreaming start to make sense – in terms of a free-running inference machine that is untethered from the sensorium.

## CONCLUSION

Conceiving of the mind as a physical force – tied to the structure and function of the brain – is not novel but giving it a detailed instantiation formalizes its scientific status. We have suggested that many of the formal properties of dreams (e.g., internally generated visual imagery, delusional beliefs about the waking state, cognitive deficits in self-reflective awareness, disorientation, impaired volition, recent memory loss, and hyper-emotionality) are delirium, by definition. If the explanations on offer are true, one can now supply the cellular and chemical basis for this (entirely normal) delirium. In doing so, we move the conscious states of dreaming and waking into their long-sought relation to brain activity. Virtual reality dream theory does not have to solve the brain–mind problem, but may contribute to the solution. This contribution raises interesting questions for example; is REM sleep necessary for consciousness?

According to the arguments in this paper, REM sleep enables the optimisation (complexity minimisation) of deep hierarchical models and may therefore be necessary for the conscious (inference) processes that are unique to deep models. Having said this, REM sleep is clearly not necessary for the emergence of embodied virtual reality models in species that do not evidence REM sleep and – to the extent that these species are conscious – it is not necessary for consciousness. Perhaps answers to these sorts of questions are not as important as the fact that these questions can be addressed formally, using the notion of hierarchical inference and virtual reality models that are grounded in neurobiological and evolutionary processes.

In short we have a mechanistic account of processes underlying conscious and unconscious inference, where this mechanism is fully embedded in a causal nexus of neuronal machinery. Potential beneficiaries of this account are our concepts of psychopathology; for example, psychosomatic disorders. If the mind is a causal physical force, then mental states can be orderly (as in physical and mental health) or disorderly (as in physical and so-called mental disease). Many have assumed that this must be so – and indeed have fruitfully pursued predictive coding in this context (Fletcher and Frith, 2009; Edwards et al., 2012; Adams et al., 2013b; Hohwy, 2013). The general point that we wish to make is that clinical psychology and psychiatry are in a position to change – and arm themselves with insights from sleep and dream science.

Clearly, there are many fascinating issues that we have not considered – issues that could be pursued from the Bayesian brain perspective offered in this article. One intriguing issue (noted by our reviewers) is the role of prediction in active inference: in other words, the role of hierarchical inference in prescribing predictions about how we will move or what we will do next. Currently, we have just focused on perceptual inference and making sense of the sensorium. It is intriguing to consider that dreaming and model optimization may also apply to inferences about our actions and how we sample outcomes from the world during wakefulness.

## Conflict of Interest Statement

The reviewer Jim Hopkins declares that, despite being affiliated to the same institution as the author Karl Friston, the review process was handled objectively and no conflict of interest exists. The authors declare that the research was conducted in the absence of any commercial or financial relationships that could be construed as a potential conflict of interest.
